# Amiodarone-Induced Myxedema Coma

**DOI:** 10.7759/cureus.9902

**Published:** 2020-08-20

**Authors:** Armaghan Raeouf, Siddarth Goyal, Jeremy Traylor

**Affiliations:** 1 Department of Medicine, Lake Erie College of Osteopathic Medicine, Erie, USA; 2 Department of Emergency Medicine, Grandview Medical Center, Dayton, USA

**Keywords:** hypothyroidism, levothyroxine, myxedema coma, amiodarone

## Abstract

Amiodarone is a class III antiarrhythmic drug often used to treat supraventricular and ventricular arrhythmias with high efficacy. Amiodarone is associated with thyroid dysfunction, which can lead to myxedema coma (MC) in undiagnosed cases. Amiodarone-induced MC is a life-threatening condition that presents a complex diagnostic challenge to emergency physicians. A 71-year-old male with a past medical history of congestive heart failure presented unresponsive to the emergency department with bradycardia and syncope. His medications included amiodarone. Work-up showed hypothermia, thyroid-stimulating hormone (TSH) of 52.2 uIU/mL, and low free T_4_ of 0.64 ng/dL. This case suggests the importance of thyroid panels in the management of patients who are using amiodarone long-term. This case also highlights a simple and effective treatment for amiodarone-induced MC.

## Introduction

Amiodarone is a class III antiarrhythmic drug often used to treat supraventricular and ventricular arrhythmias with high efficacy. It is also known to further benefit patients suffering from left ventricular systolic dysfunction due to its minimal negative inotropic action [1]. Chronic use of amiodarone can lead to several adverse effects involving various organ systems including the thyroid. Thyrotoxicity is a potential risk due to the drug’s highly iodinated chemical structure. Amiodarone-induced thyroid dysfunction is shown to occur in 15%-20% of patients taking amiodarone, irrespective of prior thyroid status [2]. Amiodarone-induced thyrotoxicosis has been suggested to cause de novo cases of hypothyroidism in which many undiagnosed cases lead to myxedema coma (MC). However, there is limited literature reporting amiodarone-induced MC, the treatment for which remains controversial [3,4].

Early-onset de novo hypothyroidism can emerge due to excessive iodine intake [5]. While it has been suggested that the thyroid can adapt to short term excess iodine, the long-term effects lead to reduced hormonal output [5-8]. Amiodarone contains 75,000 μg of Iodine per 200 mg. Consequentially, long term use of amiodarone puts patients at risk of amiodarone-induced MC.

MC is understood as hypothyroidism accompanied by physiological decompensation. It primarily presents as a combination of severe hypothermia, altered mental status, bradycardia, anasarca, and precipitating factors. Leading precipitating factors include stress, infection, and septicemia [9,10]. It has been reported recently that the mortality rate of individuals suffering from MC is 40% [11-13]. A report from Spain presents a global incidence rate of 220,000 per year, also commonly found in women and elderly [14-15]. In treating such rare cases, it is important to understand the risk factors for the development of this life-threatening condition. Here, a case of amiodarone-induced MC in a 71-year-old male is presented.

## Case presentation

A 71-year-old male presents to the emergency department with symptomatic bradycardia and syncope. To note, he had altered mental status prior to arrival and was unresponsive upon arrival. The patient had experienced a syncopal episode in the parking lot of a restaurant after having dinner. His daughter caught him before he fell so he did not sustain a head injury. His past medical history includes atrial fibrillation, coronary artery disease, congestive heart failure, chronic kidney disease stage III, and chronic systolic heart failure (left ventricular ejection fraction 30%-35%). The patient had been prescribed to wear a LifeVest wearable cardioverter defibrillator but was described by family as non-compliant. As per emergency medical services (EMS), the patient’s heart rate was at 26 bpm and he was hypotensive. Administered atropine elicited no response. A transcutaneous pacer was placed, raising his heart rate up to 70 bpm, but showed intermittent capture with an amplitude up to 175.

In the resuscitation bay, the patient was hemodynamically unstable with a heart rate as low as 20 bpm. He showed signs of anasarca with facial swelling, 3+ pitting edema in the extremities, and a firm edematous abdomen. He was hypotensive with a systolic blood pressure of 50 mmHg and was significantly short of breath. He remained unresponsive and was intubated for airway protection. A one-liter normal saline bolus was given with minimal effect on the blood pressure. As the transcutaneous pacer continued to capture poorly, the patient began to further decompensate. The possible use of a transvenous pacer was under consideration for stabilizing the patient’s heart rate; however, isoproterenol was administered instead. The isoproterenol improved both his heart rate and blood pressure, and the transcutaneous pacer was turned off. However, it became evident that the patient was hypothermic with a body temperature of 30.6 °C. Lab results indicated significant hypothyroidism with a high TSH of 52.2 uIU/mL [normal 0.358-3.742] and low free T4 of 0.64 ng/dL [normal 0.76-1.46]. The combination of signs and symptoms strongly pointed to the case of MC.

The patient’s family confirmed that there was no family history of hypothyroidism, but reported that the patient had a 14-year history of taking 200 mg amiodarone daily for atrial fibrillation. The family also recounted a hospital admission in February 2018 for upper extremity cellulitis. The patient’s lab work noted a depressed thyroid panel with an elevated TSH of 15 uIU/mL and low free T4 of 0.64 ng/dL. Previous TSH for patient earlier that year was within normal limits. His previous hospital visits suggest cellulitis as the precipitating factor for the amiodarone-induced MC.

Once the MC diagnosis was confirmed, 75 mg IV-levothyroxine was administered with 50 mg IV-solucortef. The patient was then transferred to the ICU. Following clinical course, the patient’s heart rate and blood pressure rapidly stabilized. Levothyroxine was titrated down, and within several hours of admission, his temperature normalized at 36.3 °C. The patient was extubated soon after stabilizing. Over a clinical course of three days, the patient’s TSH normalized to 6.06 uIU/mL at which he was then transferred to a tertiary care medical center to manage his case further due to the complexity of his issues.

## Discussion

MC is often a difficult diagnosis and is highly fatal if overlooked, with a mortality rate of 40% [11-13]. Characteristic symptoms that provided diagnostic clues included hypothermia, anasarca, altered mental status, and bradycardia [12,13,15]. Based on initial observations, the differential diagnoses included MI, arrhythmia, hypoglycemia, electrolyte abnormality, renal failure, sepsis, pulmonary embolism, and hypothyroidism. However, the patient’s body temperature of 30.6 °C and altered mental status suggested MC, which was confirmed with a significantly elevated TSH and low free T4.

Of note, the precipitating factor that leads to the development of MC, in this case, was systemic stress due to upper extremity cellulitis, indicated by a previous hospital admission. It was at this time that a mildly depressed thyroid panel was observed. In hindsight, these laboratory values should have been considered a high priority as his history of amiodarone therapy can cause hypothyroidism, putting the patient at a high risk of developing MC [16]. It is important to note that signs of thyroid dysfunction via thyroid panels should be closely observed in patients who undergo chronic amiodarone therapy.

Of further clinical importance, the administration of isoproterenol significantly improved a hemodynamically unstable patient suffering from hypothyroidism. The patient’s underlying condition suggests why he remained non-responsive to atropine and showed intermittent capture with a transcutaneous pacer. A transvenous pacer was ultimately avoided by the administration of isoproterenol. This is a possible intervention to consider in the treatment algorithm in a patient with symptomatic bradycardia who is refractory to atropine.

## Conclusions

Diagnosis of MC is difficult due to its various possible causes. The signs of amiodarone-induced MC are obscure, especially when treating older patients with a significant history of heart disease. However, once detected, often via clinical symptoms and serum TSH, this disease must be immediately treated with thyroid hormone. In the presenting case, MC was successfully treated with levothyroxine and glucocorticoids. Outpatient follow-ups are needed to determine the prognosis of amiodarone-induced MC.
